# Glutathionylation of Pea Chloroplast 2-Cys Prx and Mitochondrial Prx IIF Affects Their Structure and Peroxidase Activity and Sulfiredoxin Deglutathionylates Only the 2-Cys Prx

**DOI:** 10.3389/fpls.2017.00118

**Published:** 2017-01-31

**Authors:** Aingeru Calderón, Alfonso Lázaro-Payo, Iván Iglesias-Baena, Daymi Camejo, Juan J. Lázaro, Francisca Sevilla, Ana Jiménez

**Affiliations:** ^1^Department of Stress Biology and Plant Pathology, Centre for Applied Soil Science and Biology of the Segura – Consejo Superior de Investigaciones CientíficasMurcia, Spain; ^2^Department of Biochemistry, Cellular and Molecular Biology of Plants, Zaidin Experimental Station – Consejo Superior de Investigaciones CientíficasGranada, Spain

**Keywords:** 2-Cys peroxiredoxin, glutathione redox state, glutathionylation, peroxiredoxin IIF, post-translational modification, reactive nitrogen species, reactive oxygen species, sulfiredoxin

## Abstract

Together with thioredoxins (Trxs), plant peroxiredoxins (Prxs), and sulfiredoxins (Srxs) are involved in antioxidant defense and redox signaling, while their regulation by post-translational modifications (PTMs) is increasingly regarded as a key component for the transduction of the bioactivity of reactive oxygen and nitrogen species. Among these PTMs, *S*-glutathionylation is considered a protective mechanism against overoxidation, it also modulates protein activity and allows signaling. This study explores the glutathionylation of recombinant chloroplastic 2-Cys Prx and mitochondrial Prx IIF from *Pisum sativum*. Glutathionylation of the decameric form of 2-Cys Prx produced a change in the elution volume after FPLC chromatography and converted it to its dimeric glutathionylated form, while Prx IIF in its reduced dimeric form was glutathionylated without changing its oligomeric state. Mass spectrometry demonstrated that oxidized glutathione (GSSG) can glutathionylate resolving cysteine (Cys^174^), but not the peroxidatic equivalent (Cys^52^), in 2-Cys Prx. In contrast, GSSG was able to glutathionylate both peroxidatic (Cys^59^) and resolving (Cys^84^) cysteine in Prx IIF. Glutathionylation was seen to be dependent on the GSH/GSSG ratio, although the exact effect on the 2-Cys Prx and Prx IIF proteins differed. However, the glutathionylation provoked a similar decrease in the peroxidase activity of both peroxiredoxins. Despite growing evidence of the importance of post-translational modifications, little is known about the enzymatic systems that specifically regulate the reversal of this modification. In the present work, sulfiredoxin from *P. sativum* was seen to be able to deglutathionylate pea 2-Cys Prx but not pea Prx IIF. Redox changes during plant development and the response to stress influence glutathionylation/deglutathionylation processes, which may represent an important event through the modulation of peroxiredoxin and sulfiredoxin proteins.

## Introduction

The redox state of plant thiols and the regulation of cysteinyl residues in proteins are emerging as key players in the response of plants to both biotic and abiotic stresses (Sevilla et al., 2015a), functioning in redox sensing and signal transduction pathways. Reactive oxygen and nitrogen species (ROS/RNS) are known to act as signaling molecules in the maintenance of physiological functions and in the response to changing environments (Considine and Foyer, 2014). Plants are particularly exposed to oxidative and nitrosative stress, mainly due to their photosynthetic and respiratory metabolism, which can generate high levels of ROS/RNS under certain stress conditions (Martí et al., 2011; Lázaro et al., 2013). In the redox signaling process, protein thiols play a central role and redox-sensitive cysteines undergo a variety of post-translational modifications, including *S*-nitrosylation and glutathionylation, which are considered as an interesting point of control in the regulation of the protein structure and function (Hartl and Finkemeier, 2012; Zaffagnini et al., 2012a). Protein glutathionylation constitutes a reversible covalent post-translational modification (PTM) that takes place through the addition of glutathione to the thiolate of cysteines in target proteins. This modification is involved in many physiological processes, one of the most important being related to signaling, not only following oxidative or nitrosative stress but also under physiological, when thiolating agents are generated (Dalle-Donne et al., 2007). Another interesting aspect related to PTMs is the possible role as a mechanism for protecting proteins against modifications such as overoxidation (Roos and Messens, 2011). In more reducing conditions, deglutathionylation occurs as a result of the removal of the glutathione moiety from the protein, a process controlled by glutaredoxins (Grxs) and involving GSH and NADPH-dependent glutathione reductases (Meyer et al., 2012; Waszczak et al., 2015). Reversible protein glutathionylation is increasingly seen therefore not only as a major antioxidant defense against oxidative stress, but also as a cellular regulatory mechanism in cell signaling (Mieyal et al., 2008). In this context, ROS have been described as inducers of *S*-glutathionylation; more specifically, H_2_O_2_ plays an important role through its influence on the GSH/GSSG ratio, and is directly involved in the glutathionylation reaction, or through the direct oxidation of protein Cys, generating a thiyl intermediate which further reacts with GSH to form a mixed disulfide (Klatt and Lamas, 2000; Grek et al., 2013).

In cellular redox biology, there is growing interest in the involvement and regulation of the thioredoxin/peroxiredoxin/sulfiredoxin (Trx/Prx/Srx) system in plant signaling under abiotic stress conditions as an important cue that influences plant growth (Sevilla et al., 2015b). Among these redox proteins, Prxs are sensitive to glutathionylation. These ubiquitous thiol peroxidases have an antioxidant function, reducing H_2_O_2_, peroxynitrite and hydroperoxides. Mammals have six Prx isoforms (I-VI) grouped in three subfamilies, namely typical 2-Cys Prx (I-IV), atypical 2-Cys Prx (V) and 1-Cys Prx (VI), with different subcellular locations. In plants, they are localized in chloroplasts, mitochondria, nuclei, peroxisomes and cytosol, and are divided into four subgroups: 2-Cys Prx, type II Prx, Prx Q and 1-Cys Prx (Dietz, 2011). Typical chloroplast 2-Cys Prx and atypical mitochondrial Prx IIF have two characteristic cysteines involved in the reduction of peroxides, namely peroxidatic cysteine (Cp) and resolving cysteine (Cr). H_2_O_2_ oxidizes Cp to its sulfenic form that reacts with the Cr to form a disulfide bond that is reduced by thioredoxin (Trx), namely Trx*f* for chloroplast 2-Cys Prx and Trx*o* for mitochondrial Prx IIF (Barranco-Medina et al., 2008; Martí et al., 2009; Pulido et al., 2010). The difference in the reaction mechanism between both Prxs is the disulfide bond - intermolecular for chloroplast 2-Cys Prx and intramolecular for mitochondrial Prx IIF (Barranco-Medina et al., 2007; Dietz, 2011). There are also differences in their oligomeric states. Both Prxs form dimers, but while 2-Cys Prx forms decamers in its reduced or overoxidized state, Prx IIF only forms hexamers in its oxidized state (Barranco-Medina et al., 2009; Lázaro et al., 2013).

In severe oxidative stress conditions, Prxs are overoxidized to the inactive sulfinic form, which Srx, an ATP-dependent reductase located in chloroplasts and mitochondria, is able to retroreduce (Biteau et al., 2003). In fact, pea chloroplastic 2-Cys Prx and mitochondrial Prx IIF have been shown to be regenerated by pea Srx, which is then reduced by Trx (Iglesias-Baena et al., 2010, 2011). Scant information exists on the regulation of redox proteins by post-translational modifications, including the glutathionylation of chloroplastic 2-Cys Prx or mitochondrial peroxiredoxin IIF, aspects that will be addressed in this paper. 2-Cys Prx glutathionylation has been studied in the cytoplasmic protein of mammals but not in its chloroplastic counterpart of plants (Park et al., 2011; Chae et al., 2012), while human sulfiredoxin has been shown to catalyze the deglutathionylation of typical human 2-Cys Prx (Findlay et al., 2006; Park et al., 2009). In this work, the glutathionylation of both typical and atypical pea 2-Cys Prx and Prx IIF proteins is studied using reduced and oxidized glutathione (GSH, GSSG) and nitrosoglutathione (GSNO). The target cysteine residues and the oligomerization pattern after the treatments, as well as any effect on the peroxidase activity of both proteins, is analzed. In addition we study the capacity of pea Srx to deglutathionylate chloroplast 2-Cys Prx and mitochondrial Prx IIF.

## Materials and Methods

### Cloning and Purification of Recombinant Proteins

Chloroplast 2-CysPrx and mitochondrial Prx IIF from pea (*P. sativum* L. cv. Lincoln grown as described in Barranco-Medina et al., 2007) were cloned without His-tag, and expressed and purified as described by Bernier-Villamor et al. (2004) and Barranco-Medina et al. (2006). The cloning, overexpression and purification of His-tagged sulfiredoxin (Srx) from pea was performed as described by Iglesias-Baena et al. (2010). Briefly, the fragment of cDNA encoding the mature proteins was obtained by reverse transcription-PCR and cloned into the pGEM-T (Promega, Madison, USA) (2-CysPrx) or pET3d (Novagen) (PsPrx IIF) or pETM-11 (PsSrx) expression vectors. *Escherichia coli* BL21 (DE3) strains were transformed with the resulting constructions, and recombinant protein expression was induced by the addition of 1 mM isopropylthio-β-galactoside, leaving to stand for 3 h at 37°C. The *E. coli* cells were broken with a French press, followed by ammonium sulfate precipitation between 40 and 95% (w/v). The pellet was then suspended in buffer (25 mM Tris-HCl, pH 8.0, containing 150 mM NaCl) and chromatographed by FPLC.

Protein concentration was measured according to Bradford (1976) using bovine serum albumin as standard.

### GSH, GSSG, GSNO, and SNP Treatment of Recombinant Proteins

One milliliter of 50 μM of purified recombinant protein (2-CysPrx or Prx IIF) was first reduced in 50 mM Tris-HCl pH 7.5 containing 10 mM DTT for 30 min at room temperature. The DTT excess was removed by Bio-Spin 6 gel filtration (BioRad).

For the analysis by gel filtration, 200 μL of reduced proteins preparations were incubated separately either with 5 mM GSSG at 4°C for 24 h or with 5 mM GSH, GSNO, or 750 μM SNP (sodium nitroprusside) prepared in 50 mM Tris-HCl pH 7.5 for 30 min at room temperature. After incubation, the excess of the modifying agent was removed by Bio-Spin 6 column and the samples were immediately analyzed through FPLC.

For the analysis of glutathionylation using different GSH/GSSG treatments, reduced proteins (15 μg) were incubated with different mM concentrations of GSH+GSSG as 4.987+0.0125, 4.95+0.05, 4.75+0.25, and 4.5+0.5 for 5 min at 37°C and the excess of glutathionylating agents was removed by Bio-Spin 6 gel filtration (BioRad). Also a 10 mM DTT-treated sample without any glutathionylating treatment was performed as negative control. Samples were immediately analyzed by western blot as described below, loading all the treated protein in each lane.

### Gel Filtration Analysis

After treatment with GSSG, GSH, GSNO, and SNP, proteins were analyzed by gel filtration at room temperature using a Superdex-200 HR 10/30 column (GE Healthcare) equilibrated with 50 mM Tris-HCl (pH 7.5) containing 150 mM NaCl at a flow of 0.5 mL/min. Absorbance at 280 nm was recorded and 250 μL fractions were collected. The peaks of each fraction (dimer and oligomer) were collected for mass spectrometry analysis. A calibration curve of the column was performed with albumin, chymotrypsinogen, ferritin, aldolase, ovalbumin, and ribonuclease as standards (Supplementary Figure S1).

### Mass Spectrometry Analysis

Samples after FPLC chromatography were analyzed by mass spectrometry on an UltrafleXtreme Matrix-assisted laser desorption/ionization-time-of-flight/time-of-flight (MALDI-TOF/TOF) mass spectrometer (Bruker-Daltonics) in auto-mode using FlexControl v3.4 and processed using FlexAnalysis v3.4MALDI TOF/TOF apparatus (Bruker) as described in López-Vidal et al. (2016). Theoretical digestions were performed considering glutathionylation of cysteine in the peptide spectrum generated from the problem sample.

### Polyacrylamide Gel Electrophoresis and Western Blot Analysis

Denaturing SDS-PAGE was performed as described by Laemmli (1970) with acrylamide concentrations of 6% (staking gel) and 12.5% (resolving gel). Gels were stained with Coomassie Brilliant Blue R-250. For Western blot, proteins were transferred onto a nitrocellulose membrane by electroblotting. Pounceau S stained membranes were used as loading controls (Salinovich and Montelaro, 1986). Immunoreaction was performed with polyclonal antibodies against PsPrx IIF (1:3000) (Barranco-Medina et al., 2007), pea Srx peptide (CHRYEAHQKLGLPTI) (1:500) (Iglesias-Baena et al., 2010), pea 2-Cys Prx (1:5000) (Bernier-Villamor et al., 2004), and monoclonal anti-glutathione (1:500, Santa Cruz Biotechnology) diluted in TBS containing 1% (w/v) of BSA and 0.1% (v/v) of Tween-20. Anti-rabbit conjugated to alkaline phosphatase (1:7500, Boehringer Mannheim, Germany) and anti-mouse conjugated to peroxidase (1:5000, Santa Cruz Biotechnology) were used as secondary antibodies and the antigen was detected using the ECL-2 system (Thermo Scientific, USA), following the manufacturer’s instructions.

### Peroxidase Activity

Recombinant 2-Cys Prx and Prx IIF (100 μg) were treated with 10 mM DTT at room temperature for 30 min. The excess of DTT was then removed by Bio-Spin 6 gel filtration (BioRad, Spain) and 50 μg of protein were treated with 5 mM GSSG and with 4 mM GSH + 1 mM GSSG at 37°C for 5 min. The excess of GSSG and GSH was removed by Bio-Spin 6 gel filtration. Treated proteins (10 μg) were incubated with 50 μM H_2_O_2_ for 10 min at 37°C and the reaction was stopped adding 2% (p/v) trichloroacetic acid (TCA). A blank of the reaction was performed using sample buffer instead of peroxiredoxin proteins, incubated with DTT, followed by gel filtration and incubated with H_2_O_2_ and stopped with TCA. H_2_O_2_ was quantified using 100 μL of the reaction mixed with 500 μL of eFOX medium according Cheeseman (2006). H_2_O_2_ was determined based on the difference in absorption at 550 nm using a standard curve that covered the range of 0–200 μM.

### Deglutathionylation of 2-Cys Prx and Prx IIF by Srx

Fifty microgram of recombinant 2-Cys Prx and Prx IIF were reduced with DTT as described above in a final volume of 50 μL and subjected to Bio-Spin 6 gel filtration. Forty microliter of these reduced proteins were incubated with 5 mM GSSG at 4°C for 24 h and dialyzed again to eliminate the excess of GSSG. A final concentration of 3 μM of GSSG-treated peroxiredoxin was incubated with a final concentration of 6 μM of DTT-treated Srx for different times and the reactions were stopped adding the non-reducing SDS-buffer. Finally, 3 μg of treated proteins were subjected to western blot analysis.

### Statistics

The results are the mean of three replicates from each experiment which were repeated at least two times. The significance of any differences between the mean values was determined by one-way analysis of variance. The Tukey’s test was used to compare the means. All error bars represent standard error (SE) of the mean. The asterisk above the bars indicates significant difference (*P* < 0.05).

## Results

### Effect of Glutathionylation on the Oligomeric State of 2-Cys Prx

**FIGURE 1:**
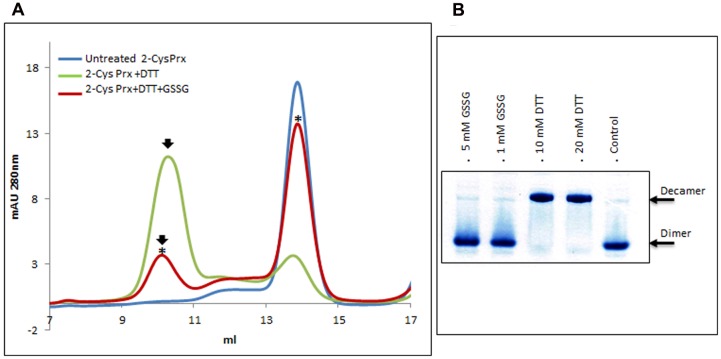
**Elution profile after size exclusion chromatography (A)** through Superdex-200 HR 10/30 column of native (untreated) recombinant pea 2-Cys Prx and after its treatment with 10 mM DTT (+DTT) and 10 mM DTT + 5 mM GSSG (+DTT+GSSG). Asterisks indicate the samples subsequently analyzed by MALDI TOF/TOF. Pattern of oligomerization **(B)** analyzed by native PAGE and Coomasie staining of pre-reduced protein treated with GSSG or DTT, the non-treated protein (-DTT, -GSSG) being used as control.

**FIGURE 2:**
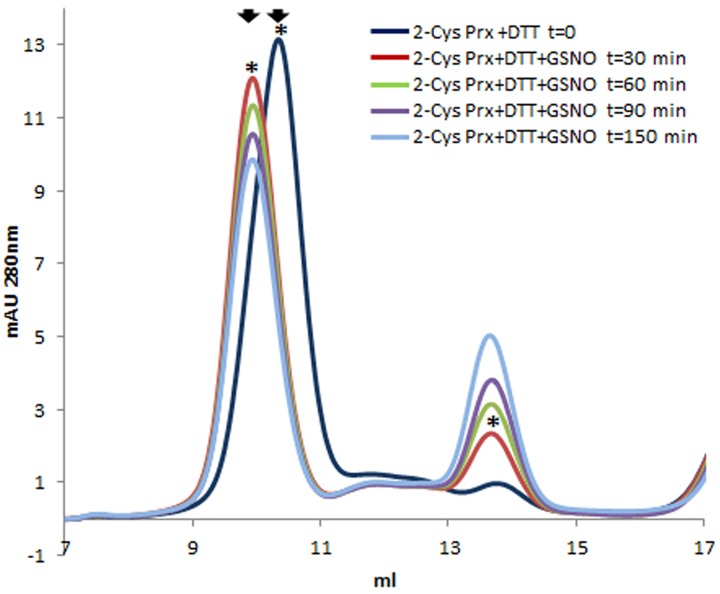
**Elution profile after size exclusion chromatography through Superdex-200 HR 10/30 column of pre-reduced pea 2-Cys Prx (+DTT) and after its treatment with 5 mM GSNO for different incubation times**. Asterisks indicate the samples subsequently analyzed by MALDI TOF/TOF.

After gel filtration on Superdex-200 HR 10/30, untreated 2-Cys Prx protein appeared exclusively as a dimer (**Figure 1A**), but when reduced with DTT, the protein mainly changed to its decameric form. Treating this reduced form with GSSG produced two modifications (**Figure 1A**): the decamer eluted earlier and it was also dissociated to a dimer. The oligomerization pattern was also analyzed by native PAGE and Coomasie staining (**Figure 1B**). The 2-Cys Prx protein pre-reduced with DTT was treated with GSSG and the protein without any reducing treatment was used as control. The treatment with 1 and 5 mM GSSG was carried out at 37°C for 5 min to check whether the changes in the oligomeric pattern also occurred in these conditions. The treatment of the DTT-treated 2-Cys Prx protein with GSNO also showed an advance in the elution volume of the decamer and its concentration fell slowly as the dimer concentration increased (**Figure 2**), the change being dependent on the incubation time.

**FIGURE 3:**
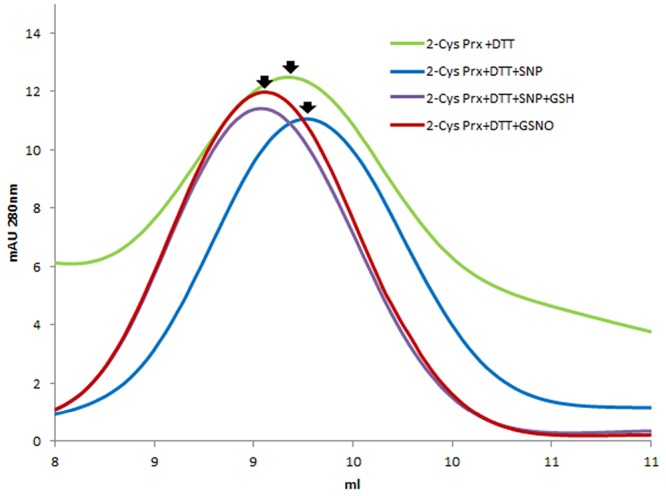
**Elution profile after size exclusion chromatography through Superdex-200 HR 10/30 column of DTT-reduced pea 2-Cys Prx and after its treatment with different *S*-nitrosylating and glutathionylating agents: 750 μM SNP, 750 μM SNP + 5 mM GSH and 5 mM GSNO**.

Mass spectrometry analysis of the decameric and dimeric forms eluted from the gel filtration of the 2-Cys Prx protein treated with both GSSG and GSNO pointed to the glutathionylation of the resolving Cys^174^ but not of the peroxidatic Cys^52^ in both oligomeric forms (**Table 1** and Supplementary Figure S2). To ascertain whether a nitrosylated form of 2-Cys Prx was susceptible to glutathionylation with GSH, the reduced protein was first treated with an *S*-nitrosylating agent, sodium nitroprusside (SNP). It was observed that the reduced decamer delayed its elution volume, unlike the glutathionylated protein that forwarded the elution volume as shown in **Figure 3**. The subsequent treatment with GSH showed a similar result to that obtained by direct modification with GSSG.

**Table 1:** Glutathionylation of purified recombinant pea 2-Cys Prx analyzed by mass spectrometry MALDI-TOF/TOF after treatment of the pre-reduced (DTT) protein (2CPSH) with 5 mM GSSG or GSNO for 30 min (See Supplementary Figure S2).

Protein	Sample	Cysteine	Modification
2-Cys-Prx	2CPSH	C^52^	NO
		C^174^	NO
	2CPGSSG Decamer	C^52^	NO
		**C^174^**	**YES** 3105.40 (Cys-SG)
	2CPGSSG Dimer	C^52^	NO
		**C^174^**	**YES** 3105.43 (Cys-SG)
	2CPGSNO Decamer	C^52^	NO
		**C^174^**	**YES** 3105.46 (Cys-SG)
	2CPGSNO Dimer	C^52^	NO
		**C^174^**	**YES** 3105.39 (Cys-SG)

*The incorporation of a SG group in the Cys^*52*^ or Cys^*174*^ in the decameric and dimeric forms of the protein eluted from the size exclusion chromatography through Superdex-200 HR 10/30 is pointed in bold*.

### Effect of Glutathionylation on the Oligomeric State of Prx IIF

**FIGURE 4:**
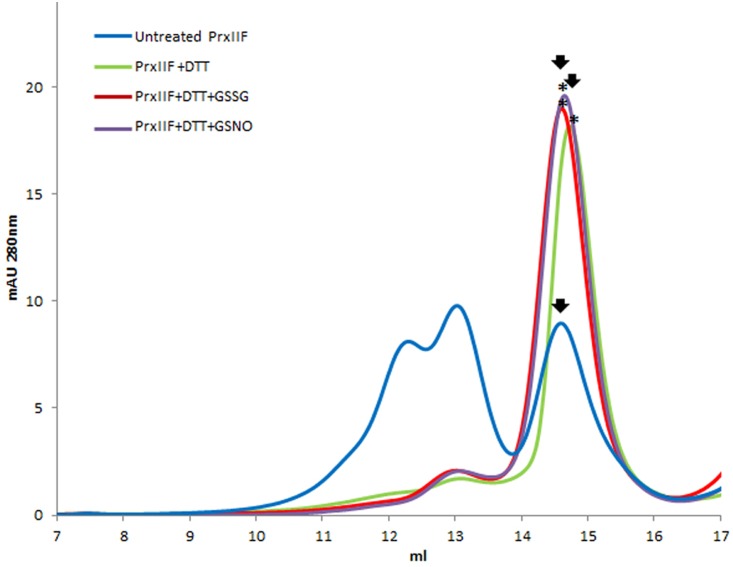
**Elution profile after size exclusion chromatography through Superdex-200 HR 10/30 column of native (untreated form) recombinant pea Prx IIF and after its treatment with 10 mM DTT and 5 mM GSSG and GSNO**. Asterisks indicate the samples subsequently analyzed by MALDI TOF/TOF.

The reduction of Prx IIF by DTT provoked a change in the hexameric form of the protein to a dimeric one. Glutathionylation of the dimeric form with both GSSG and GSNO, showed a slight shift (advance) in its elution volume (**Figure 4**). In this case, mass spectrometry analysis pointed to the glutathionylation of both resolving and peroxidatic Cys (59 and 84) (**Table 2** and Supplementary Figure S3).

**Table 2:** Glutathionylation of purified recombinant pea Prx IIF analyzed by mass spectrometry MALDI-TOF/TOF after treating the pre-reduced (DTT) protein (IIFSH) with 5 mM GSSG or GSNO for 30 min.

Protein	Sample	Cysteine	Modification
Prx IIF	IIFSH	C^59^	NO
		C^84^	NO
	IIFGSSG	**C^59^**	**YES** 2002.94 (Cys-SG) 2131.06 (Cys-SG)
		**C^84^**	**YES** 2669.20 (Cys-SG) 2868.33 (Cys-SG)
	IIFGSNO	**C^59^**	**YES** 2002.96 (Cys-SG) 2131.04 (Cys-SG)
		**C^84^**	**YES** 2669.20 (Cys-SG) 2868.33 (Cys-SG)

*The incorporation of a SG group in the Cys^*59*^ or Cys^*84*^ in the dimeric form of the protein eluted from the size exclusion chromatography through Superdex-200 HR 10/30 is pointed in bold*.

### Effect of Different GSH/GSSG Treatments on the Glutathionylation of 2-Cys Prx and Prx IIF

**FIGURE 5:**
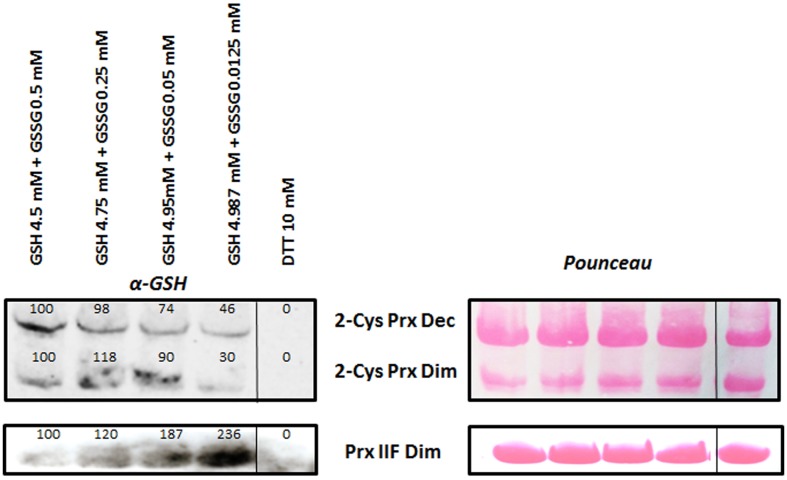
**Glutathionylation of recombinant 2-Cys Prx and Prx IIF by different concentrations of GSH/GSSG**. DTT-reduced Prx proteins were incubated with the different concentrations for 5 min at 37°C and the excess of glutathionylating agents was removed. A DTT-treated sample was used as negative control. Samples (15 μg protein) were immediately analyzed by western-blot using a specific monoclonal glutathione antibody, and a representative example is shown. Numbers show the mean of the densitometric analysis of at least 4 independent experiments, relative to the first band in each of the forms of the proteins. Pounceau S stained membranes were used as loading controls. Dec: decameric and Dim: dimeric forms of the proteins.

DTT-reduced recombinant proteins (15 μg) were treated with different GSH+GSSG mM concentrations (4.987+0.0125, 4.95+0.05, 4.75+0.25, and 4.5+0.5) and a 10 mM DTT sample without any glutathionylating treatment was used as negative control of the experiment. As shown in **Figure 5**, increasing concentration of GSSG increased glutathionylation of both the oligomeric and dimeric forms of 2-Cys Prx. However, the opposite was observed for the Prx IIF, the glutathionylation of which decreased as GSH diminished, although in general, a higher signal of glutathionylation was observed for this protein compared to that of the 2-Cys Prx. A representative gel is shown for each protein and numbers above the signals represent the mean of the densitometric analysis of the bands corresponding to four independent experiments. Pounceau staining was used to correct the loading.

### Effect of Glutathionylation on the Peroxidase Activity

**FIGURE 6:**
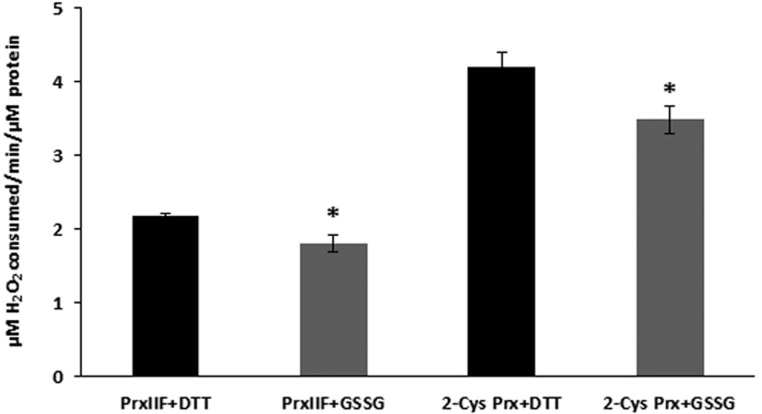
**Peroxidase activities of recombinant 2-Cys Prx and Prx IIF after the treatment with 5 mM GSSG**. Peroxidase activity was measured in previously DTT-reduced proteins (control) and in GSSG-treated proteins after incubation with H_2_O_2_ for 10 min at 37°C, using trichloroacetic acid to stop the reactions, as described in material and methods. H_2_O_2_ was then quantified using the eFOX method.

Glutathionylation of 2-Cys Prx and Prx IIF led to a reduction in the peroxidase activity of the proteins, as represented by the H_2_O_2_ consumed in the reaction. This activity was measured in the DTT-reduced proteins (control) and after the treatment of the proteins with GSSG, as described above (**Figure 6**). The activity was found to be similarly reduced by the glutathionylation treatment, the reduction being approx 17% for both peroxiredoxin proteins. Similar results were found for a 4 mM GSH + 1 mM GSSG treatment (data not shown).

### Deglutathionylation of Glutathionylated Proteins by Srx

**FIGURE 7:**
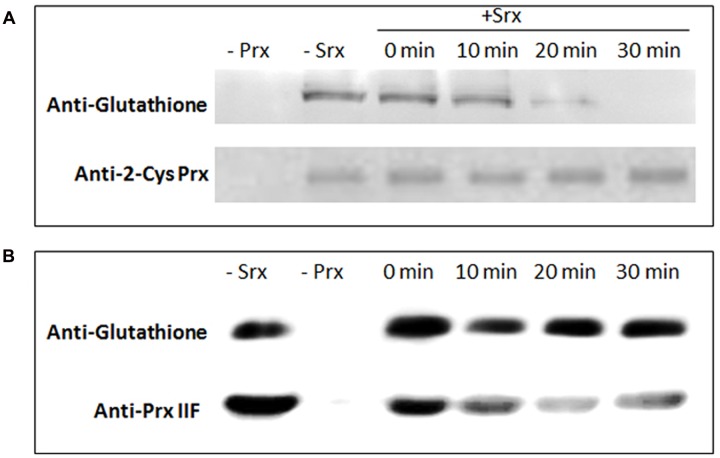
**Deglutathionylation of recombinant 2-Cys Prx (A)** and Prx IIF **(B)** proteins by pea recombinant Srx. Prx proteins were treated with 5 mM GSSG and then incubated for different times with recombinant DTT-treated Srx. The samples (3 μg of protein) were analyzed by western-blot using a monoclonal glutathione antibody. The loading was checked using specific polyclonal 2-Cys Prx and Prx IIF antibodies.

To assess whether the pea Srx protein was able to deglutathionylate both pea 2-Cys Prx and Prx IIF glutathionylated proteins, the proteins were first treated with GSSG and then both peroxiredoxins were incubated for different times with recombinant pea Srx protein previously reduced with DTT. After analysis of all the treated proteins by western blot using a specific GSH antibody, the deglutathionylation of 2-Cys Prx was found to be catalyzed by Srx, and the decrease in the glutathionylation was detected after 10 min (**Figure 7A**). However, Srx did not deglutathionylate Prx IIF in the analyzed conditions (**Figure 7B**). The loading was checked using a specific polyclonal 2-Cys Prx and Prx IIF antibodies.

## Discussion

Peroxiredoxins in non-photosynthetic organisms have been described as being among the proteins that are modified by the redox-sensitive mechanisms of glutathionylation and deglutathionylation, which, along with other post-translational mechanisms, are known to regulate their function, allowing localized H_2_O_2_ to build up, as described in the floodgate model (reviewed by Chae et al., 2012; Sevilla et al., 2015a). In plants, there is scarce information about glutathionylated proteins, although a 2 Cys-Prx has been described in *Arabidopsis* as being one of the proteins targets of this PTM (Dixon et al., 2005). Changes in the oligomeric pattern have been described as a consequence of PTMs of several proteins including peroxiredoxins from different origins, although the effect on chloroplastic 2-Cys Prx or mitochondrial Prx IIF is unknown. The transition from decamers to dimers of chloroplastic 2 Cys Prx due to glutathionylation, as observed in this study after treatment with GSSG and GSNO, is similar to that described for human cytosolic Prx I (Park et al., 2009, 2011). These authors reported that three of the four Prx I cysteine residues, Cys52, Cys83, and Cys173, were glutathionylated when treated with GSSG. Moreover, the glutathionylation of 50 μM Prx I was shown to promote changes in its quaternary structure from decamers (representing 97% of the total reduced protein) to mainly dimers with a higher peroxidase activity. This modification also provoked the inactivation of its molecular chaperone function mainly through the glutathionylation of a Cys^83^ located at the dimer–dimer interface and probably involved in the stabilization of the decamers (Chae et al., 2012). In this way, glutathionylation is able to alter the structure and thus the function of this antioxidant protein (Park et al., 2011), with possible implications in situations involving redox changes or oxidative/nitrosative stress. On the other hand, human Prx II, another cytosolic 2-Cys Prx which lacks Cys^83^, has been described as being less susceptible to glutathionylation by glutathione than Prx I, which may not be easily accessible to interact with the peroxidatic and resolving Cys^52^ and Cys^173^ in the dimer (Park et al., 2011). The chloroplast 2-Cys Prx studied in the present work has two cysteines, Cys^52^ and Cys^174^, the latter resolving Cys being glutathionylated. The observed change in the elution profile of the decamer after FPLC Superdex-200 HR 10/30 chromatography of the glutathionylated protein could be due to a conformational change, because the possible change in molecular mass as a result of the addition of GSH would not seem to justify this behavior. On the other hand, glutathionylation of this Cys seems to destabilize the decamer and the protein is present mainly as a dimer. Any structural change in Prxs may affect their redox state, oligomeric structure, and/or interaction with other proteins and could have a significant impact on the cascade of H_2_O_2_-related signaling events (Sevilla et al., 2015a). It has been described that functional 2-Cys Prx is a dimer (Dietz, 2011) and we have found that glutathionylation of both the decamer and dimer forms negatively affected peroxidase activity strongly suggesting that glutathionylation affects the function of this protein in chloroplasts. This is especially interesting taking into account the recent suggestion concerning the chaperone function of plant 2-Cys Prx, which does not seem to be essential in planta, because of the absence of high-molecular weight complexes under severe but physiological water deficit and photooxidative stress conditions, highlighting the peroxidase activity of this protein (Cerveau et al., 2016).

As regard PsPrx IIF, glutathionylation did not induce a change in its oligomeric state but produced a similar shift in the elution profile to that recently described for *S*-nitrosylation of the protein after GSNO and SNP treatments: an advance in the elution volume of both the hexameric and dimeric forms (Camejo et al., 2015). While the effect of *S*-nitrosylation was described as decreasing the peroxidase activity of Prx IIF, which acquired a new transnitrosylase activity on its target protein, citrate synthase, the effect of glutathionylation of this protein has not been evaluated before. The decreased peroxidase activity found following the glutathionylation of both Cys residues in Prx IIF by GSNO and GSSG points to an additional post-translational modification of this peroxidase in the mitochondria that would influence its role in redox control, with potential implications for cell signaling.

Protein glutathionylation is primarily influenced by the glutathione redox state and the most studied mechanism of glutathionylation is the spontaneous thiol/disulfide exchange between GSSG and a protein cysteine thiol (Zaffagnini et al., 2012b). Reduced glutathione concentration in organelles such as chloroplasts and mitochondria are described to be around 1-5 mM and 6-10 mM, respectively (Foyer and Halliwell, 1976; Law et al., 1983; Bielawski and Joy, 1986; Noctor et al., 1998; Koﬄer et al., 2013). The glutathione pool is kept highly reduced by glutathione reductase and the relationship between GSH and GSSG are usually in the range of 95% GSH 5% GSSG, although Zaffagnini et al. (2012b) described a GSH/GSSG ratio of around 10^5^. Moreover, these authors described that a change in the GSH/GSSG to 1 (K_ox_ of the Cys: a value that is thought to be the range at which 50% of the proteins could be glutathionylated), was unlikely to have occurred during stress. However, a chloroplastic GRX of poplar has a K_ox_ of 309 and might be glutathionylated *in vivo* under physiological stress conditions (Couturier et al., 2009). On the other hand, it has been reported that the GSH/GSSG ratio influences the extent of oxidation of mitochondrial protein thiols by GSSG (Beer et al., 2004). Thus, in order to determine whether the glutathionylation of 2-Cys Prx and Prx IIF was dependent on this ratio, the assays were performed in the presence of different GSH/GSSG ratios, and the results were different for the two proteins, with an increase in the glutathionylation of the 2-Cys Prx and Prx IIF dependent on increasing concentrations of GSSG and GSH, respectively, with a higher amount of PrxIIF protein being glutathionylated in the assayed conditions. This different behavior is interesting taking into account the different subcellular location of both peroxiredoxins and the different susceptibilities of the Cys to be glutathionylated, as well as the different effect on the oligomerization of the proteins. *S*-glutathionylation seems to be dependent on the different sensitivity of the Cys residues to the glutathionylating agent although further studies will be necessary to determine the exact mechanism underlying this different effect. Anyway, it is important to point out that very small changes in GSH/GSSG during cellular metabolism but mainly during stress could play a key role in signaling events, while the fact that 2-Cys Prx and Prx IIF thiol glutathionylation was sensitive to these changes points to a fine regulation by a mild oxidation of the glutathione pool. In light of the rapid response to this PTM, both peroxiredoxins may contribute to the antioxidant defense in chloroplasts and mitochondria, regulating H_2_O_2_ concentration and signaling.

**FIGURE 8:**
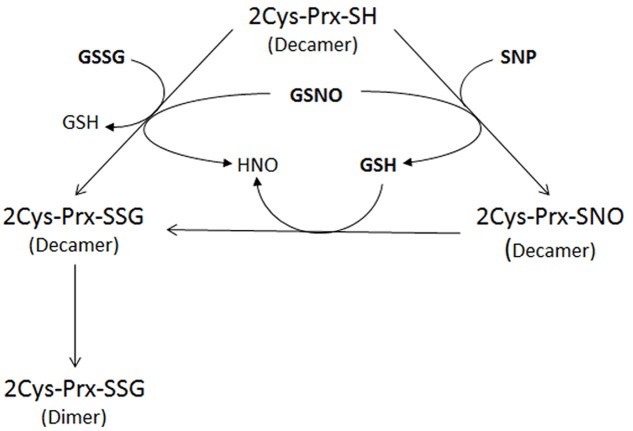
**Proposed mechanism of glutathionylation of the decameric reduced form of 2-Cys Prx after treatment with 5 mM GSSG, 5 mM GSNO or 750 μM SNP**. Glutathionylation of the 2-Cys Prx can be caused directly by GSSG or GSNO or indirectly by GSH after nitrosylation of the protein by SNP. The glutathionylation of the decameric 2-Cys Prx induces dimerization of the protein.

The interplay between nitrosative and oxidative stress could be through PTMs which may lead to a conformational change in the proteins that could prevent their overoxidation or carbonylation and thus the irreversible loss of function. This point of control might be especially important for an adequate plant response (Tanou et al., 2009; Camejo et al., 2013). More specifically an interplay between *S*-nitrosylation and *S*-glutathionylation exists, the physiological agent GSNO being able to produce both modifications. As an example, human eNOS has been described as being regulated by glutathionylation (Chen et al., 2010). To check whether a nitrosylated form of 2-Cys Prx could also be glutathionylated, the protein was first treated with an *S*-nitrosylating agent (SNP) before glutathionylation with GSH. The result was similar to that obtained with the direct treatment with GSNO, suggesting that glutathionylation of the decamer could be caused directly by GSSG or GSNO or indirectly by GSH on a nitrosylated form of the protein (**Figure 8**); in fact, GSH has been seen to glutathionylate –SOH or -SNO groups (Zaffagnini et al., 2012b). Once glutathionylated, the decamer would change to the dimeric form, probably as a result of the conformational instability of the decameric glutathionylated form. It has been described that NO-induced GSH oxidation may contribute to RNS-induced protein thiolation. The reaction of GSH with protein thiols that are *S*-nitrosylated upon exposure to RNS converts nitrosylated cysteines into relatively more stable mixed disulfides (Klatt and Lamas, 2000). On the other hand, many of the proteins reported as *S*-thiolated under oxidative conditions have subsequently been described as being susceptible to the formation of a mixed disulfide in response to RNS. Therefore the *S*-glutathionylation of active-site cysteines may integrate oxidative and nitrosative stress via redox-dependent and/or redox-independent (through GSNO) mechanisms (Klatt and Lamas, 2000).

Different results have been attributed to the capacity of Srx to deglutathionylate many protein targets *in vitro* and *in vivo* following oxidative and/or nitrosative stress (Findlay et al., 2006), including Prxs, depending on the glutathionylating agent. In the present work, plant sulfiredoxin deglutathionylated GSSG-treated 2-Cys Prx and not Prx IIF. Human Srx has also been described to deglutathionylate GSSG-treated Prx I, a ubiquitous 2-Cys Prx, but not Prx V, an atypical 2-Cys Prx (like plant Prx IIF). It has been described that Cys^83^ and Cys^173^ residues were preferentially deglutathionylated by Srx, and glutathionylated Srx was found as intermediate, which was rapidly deglutathionylated by GSH, whereas glutaredoxin I deglutathionylated Cys^52^ (Park et al., 2009). The fact that pea chloroplastic/mitochondrial Srx is not able to deglutathionylate Prx IIF implies that glutaredoxin might be a potential key protein in the mitochondria and thus in ROS/RNS functionality in this organelle, an aspect that merits further attention, while in chloroplast, the Srx protein could play a central role in the redox control. Both proteins would be involved in cell signaling in oxidative or nitrosative environments as a result of their glutathionylation/deglutathionylation, which may influence protein function, affecting among others, the H_2_O_2_ or hydroperoxide levels.

## Conclusion

The glutathionylation of pea chloroplastic 2-Cys Prx and mitochondrial Prx IIF induced a change in their structure but also in the oligomerization state of the chloroplastic enzyme. The peroxidase activity of both proteins was similarly reduced by glutathionylation, which was detected in the resolving cysteine of 2-Cys Prx and in both Cys of the Prx IIF protein. Glutathionylation was dependent on the GSH/GSSG ratio, which affected both proteins differently, and sulfiredoxin was able to deglutathionylate 2-Cys Prx but not Prx IIF. In this way, glutathionylation may act, on the one hand, as a temporary protection of peroxiredoxins in physiological processes in which oxidative and/or nitrosative stress are involved and, on the other hand, this PTM could play a significant role in situations where H_2_O_2_ acts as a signaling molecule, modulating the peroxidase activity of these proteins. Studies of the biological relevance of the glutathionylation-deglutathionylation processes *in vivo* for peroxiredoxin proteins and their involvement during plant development or stress response are being conducted in order to establish the significance of these modifications.

## Author Contributions

JL, FS, and AJ designed research. AC, AL-P, II-B, and DC carried out research. JL, FS, and AJ wrote and revised the manuscript. All authors discussed the results and commented the manuscript and have given approval to the final version of the manuscript.

## Conflict of Interest Statement

The authors declare that the research was conducted in the absence of any commercial or financial relationships that could be construed as a potential conflict of interest.
