# Supplementary material for: Glutathionylation of Pea Chloroplast 2-Cys Prx and Mitochondrial Prx IIF Affects Their Structure and Peroxidase Activity and Sulfiredoxin Deglutathionylates Only the 2-Cys Prx
Source: Front Plant Sci. 2017 Jan 31;8:118. doi: 10.3389/fpls.2017.00118 (PMC5283164; doi:10.3389/fpls.2017.00118)
Supplement: Supplementary file 1 [file Presentation_1.PDF]

Supplementary Material

The effect of glutathionylation on structure and activity of pea (*Pisum sativum* L.) chloroplast 2-Cys Prx and mitochondrial Prx IIF and deglutathionylation by sulfiredoxin

Aingeru Calderón[1*], Alfonso Lázaro-Payo[2*], Iván Iglesias-Baena[2], Daymi Camejo[1], Juan J. Lázaro[2], Francisca Sevilla[1] and Ana Jiménez[1ꙴ]

*These two authors contributed equally to this work

ꙴ **Correspondence:** Ana Jiménez: ajimenez@cebas.csic.es

1 Supplementary Figures

Figure S1. Calibration curve of the Superdex-200 HR 10/30 column.

Figure S2. Mass spectrometry MALDI-TOF/TOF analysis of DTT-reduced pea 2-Cys Prx treated with 5 mM GSNO and 5 mM GSSG after separation of the decamer (Dec) and dimer (Dim) by size exclusion chromatography through Superdex-200 HR 10/30, identifying the Cys presenting the incorporation of a SG group. Mass spectrometry of DTT treated 2-Cys Prx (2CPSH) is presented as control. Samples analysed are pointed by asterisks in Figures 1 and 2.

Figure S3. Mass spectrometry MALDI-TOF/TOF analysis of DTT-reduced pea Prx IIF treated with 5 mM GSNO and 5 mM GSSG after size exclusion chromatography through Superdex-200 HR 10/30, identifying the Cys presenting the incorporation of a SG group. Mass spectrometry of Prx IIF after the 10 mM DTT treatment (IIFSH) is also showed as control. Samples analysed are pointed by asterisks in Figure 4.
