# Supplementary figures and images for: Glutathionylation of Pea Chloroplast 2-Cys Prx and Mitochondrial Prx IIF Affects Their Structure and Peroxidase Activity and Sulfiredoxin Deglutathionylates Only the 2-Cys Prx
Source: Front Plant Sci. 2017 Jan 31;8:118. doi: 10.3389/fpls.2017.00118 (PMC5283164; doi:10.3389/fpls.2017.00118)

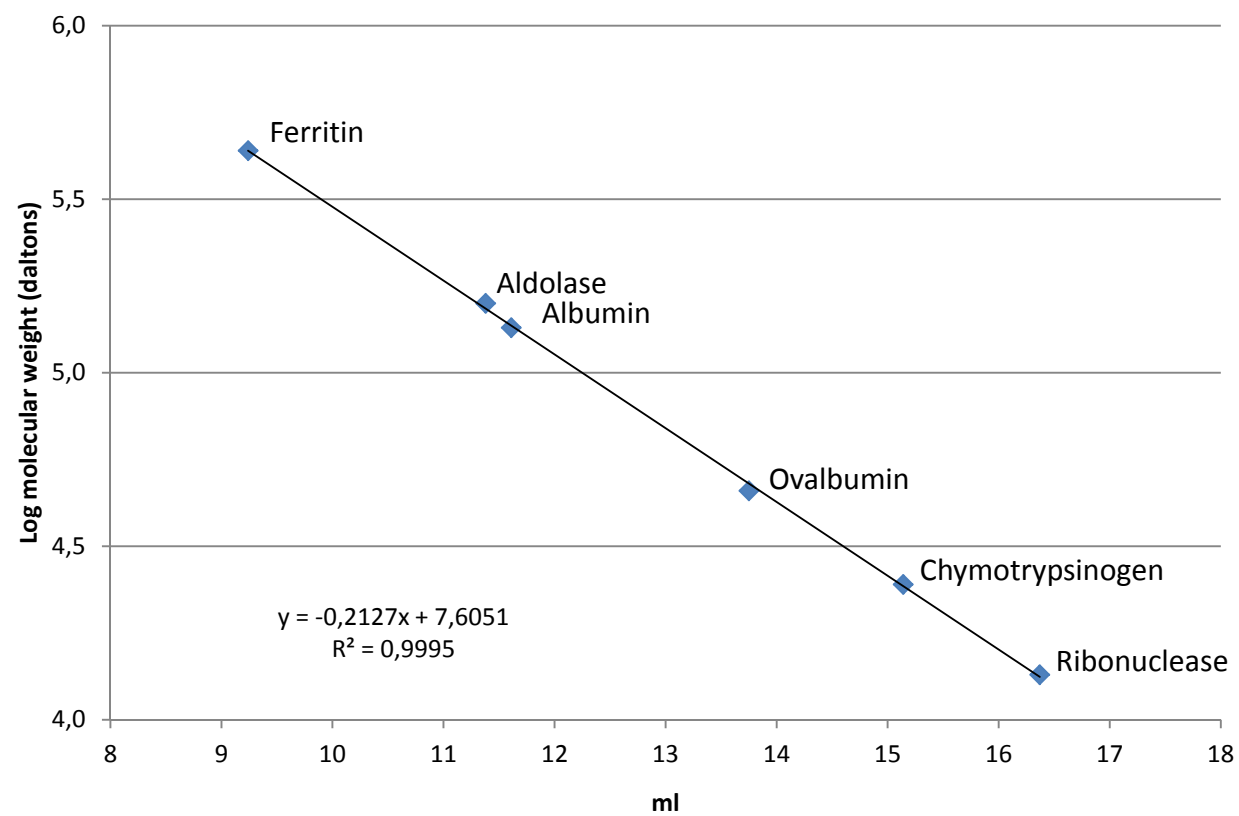

**Fig. S1.** Calibration curve of the Superdex-200 HR 10/30 column.

Supplement: Supplementary file 2 [file Presentation_2.PDF]
