# Supplementary material for: Glutathionylation of Pea Chloroplast 2-Cys Prx and Mitochondrial Prx IIF Affects Their Structure and Peroxidase Activity and Sulfiredoxin Deglutathionylates Only the 2-Cys Prx
Source: Front Plant Sci. 2017 Jan 31;8:118. doi: 10.3389/fpls.2017.00118 (PMC5283164; doi:10.3389/fpls.2017.00118)
Supplement: Supplementary file 3 [file Presentation_3.PDF]

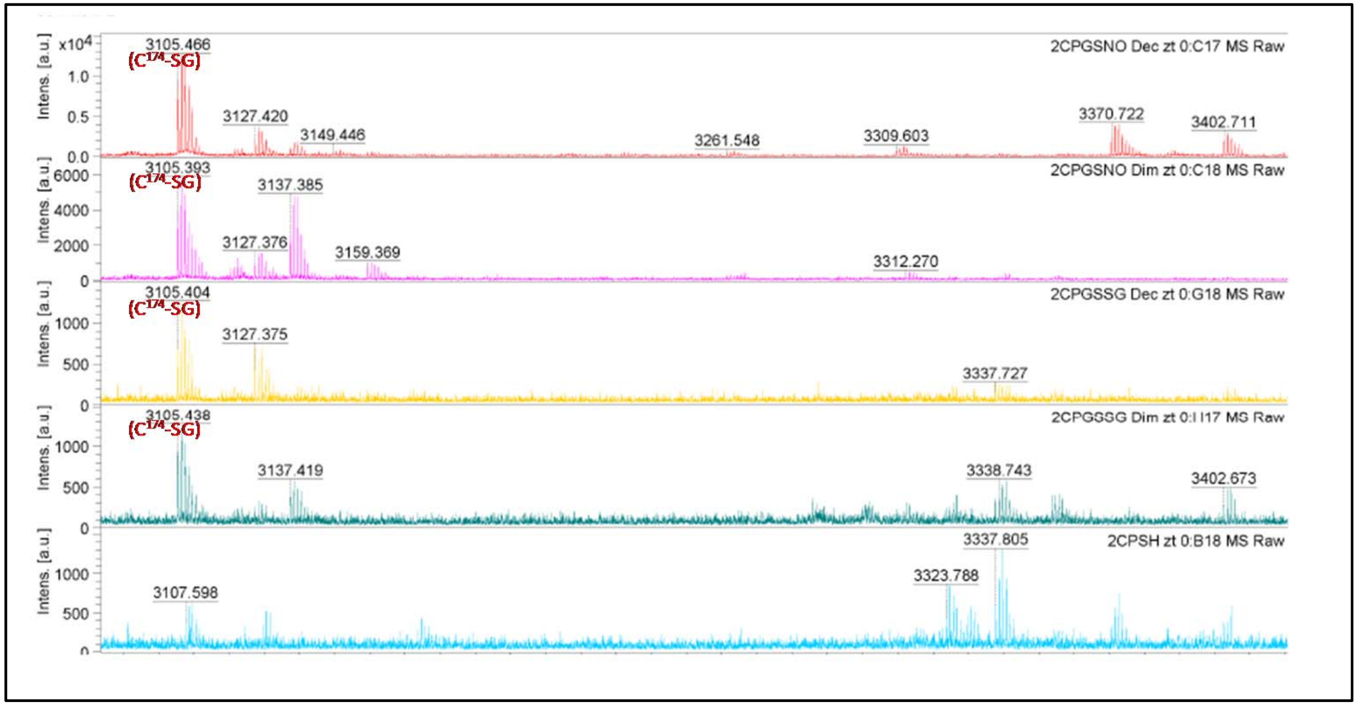

Fig. S2. Mass spectrometry MALDI-TOF/TOF analysis of DTT-reduced pea 2-Cys Prx treated with 5 mM GSNO and 5 mM GSSG after separation of the decamer (Dec) and dimer (Dim) by size exclusion chromatography through Superdex-200 HR 10/30, identifying the Cys presenting the incorporation of a SG group. Mass spectrometry of DTT treated 2-Cys Prx (2CPSH) is presented as control. Samples analysed are pointed by asterisks in Figures 1 and 2.
