# Supplementary material for: Glutathionylation of Pea Chloroplast 2-Cys Prx and Mitochondrial Prx IIF Affects Their Structure and Peroxidase Activity and Sulfiredoxin Deglutathionylates Only the 2-Cys Prx
Source: Front Plant Sci. 2017 Jan 31;8:118. doi: 10.3389/fpls.2017.00118 (PMC5283164; doi:10.3389/fpls.2017.00118)
Supplement: Supplementary file 4 [file Presentation_4.PDF]

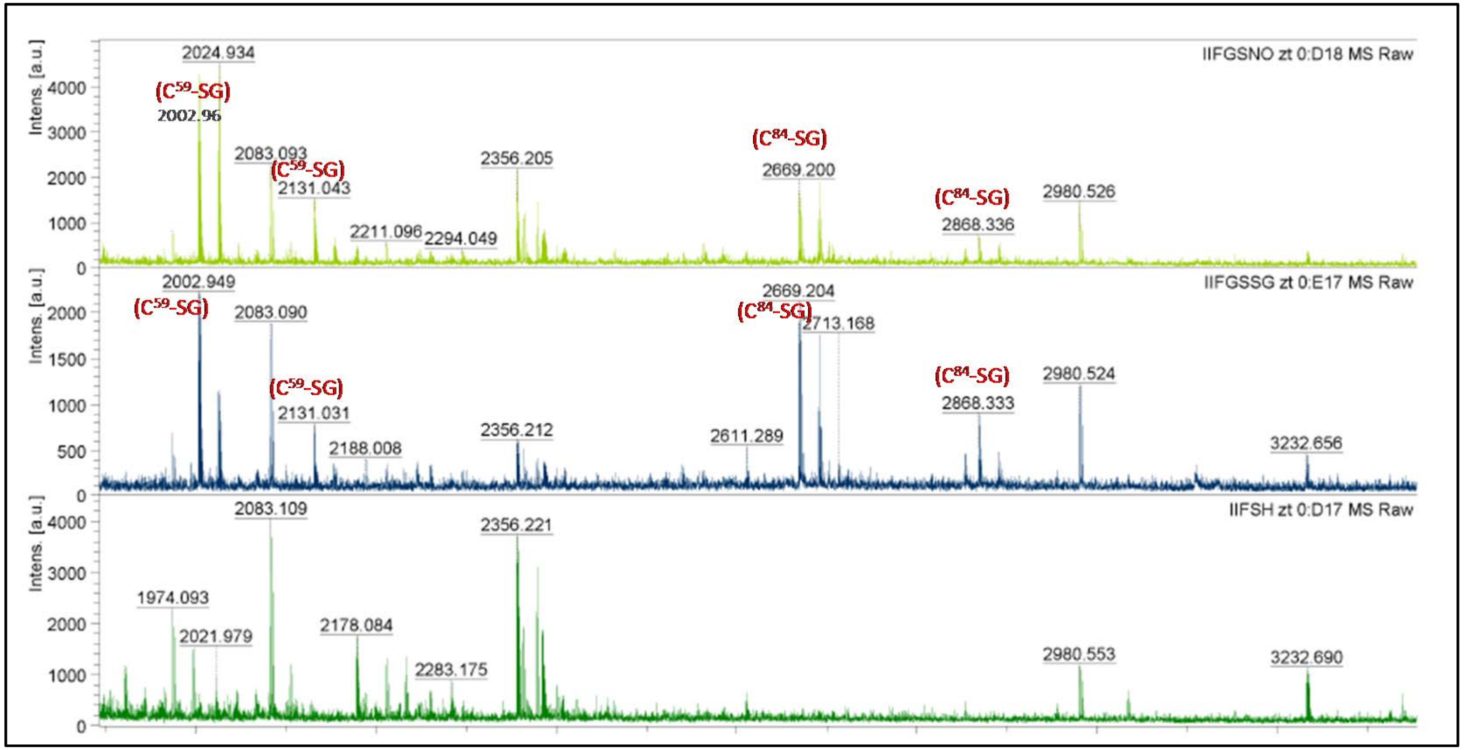

Fig. S3. Mass spectrometry MALDI-TOF/TOF analysis of DTT-reduced pea Prx IIF treated with 5 mM GSNO and 5 mM GSSG after size exclusion chromatography through Superdex-200 HR 10/30, identifying the Cys presenting the incorporation of a SG group. Mass spectrometry of Prx IIF after the 10 mM DTT treatment (IIFSH) is also showed as control. Samples analysed are pointed by asterisks in Figure 4.
